# Effects of Nitrogen and Phosphorus Fertilization on Soil Carbon Fractions in Alpine Meadows on the Qinghai-Tibetan Plateau

**DOI:** 10.1371/journal.pone.0103266

**Published:** 2014-07-30

**Authors:** Jin Hua Li, Yu Jie Yang, Bo Wen Li, Wen Jin Li, Gang Wang, Johannes M. H. Knops

**Affiliations:** 1 State Key Laboratory of Grassland Agro-Ecosystems, School of Life Sciences, Lanzhou University, Lanzhou, P.R. China; 2 School of Biological Sciences, University of Nebraska, Lincoln, Nebraska, United States of America; Agricultural Research Service, United States of America

## Abstract

In grassland ecosystems, N and P fertilization often increase plant productivity, but there is no concensus if fertilization affects soil C fractions. We tested effects of N, P and N+P fertilization at 5, 10, 15 g m^−2^ yr^−1^ (N_5_, N_10_, N_15_, P_5_, P_10_, P_15_, N_5_P_5_, N_10_P_10_, and N_15_P_15_) compared to unfertilized control on soil C, soil microbial biomass and functional diversity at the 0–20 cm and 20–40 cm depth in an alpine meadow after 5 years of continuous fertilization. Fertilization increased total aboveground biomass of community and grass but decreased legume and forb biomass compared to no fertilization. All fertilization treatments decreased the C:N ratios of legumes and roots compared to control, however fertilization at rates of 5 and 15 g m^−2^ yr^−1^ decreased the C:N ratios of the grasses. Compared to the control, soil microbial biomass C increased in N_5_, N_10_, P_5_, and P_10_ in 0–20 cm, and increased in N_10_ and P_5_ while decreased in other treatments in 20–40 cm. Most of the fertilization treatments decreased the respiratory quotient (*q*CO_2_) in 0–20 cm but increased *q*CO_2_ in 20–40 cm. Fertilization increased soil microbial functional diversity (except N_15_) but decreased cumulative C mineralization (except in N_15_ in 0–20 cm and N_5_ in 20–40 cm). Soil organic C (SOC) decreased in P_5_ and P_15_ in 0–20 cm and for most of the fertilization treatments (except N_15_P_15_) in 20–40 cm. Overall, these results suggested that soils will not be a C sink (except N_15_P_15_). Nitrogen and phosphorus fertilization may lower the SOC pool by altering the plant biomass composition, especially the C:N ratios of different plant functional groups, and modifying C substrate utilization patterns of soil microbial communities. The N+P fertilization at 15 g m^−2^ yr^−1^ may be used in increasing plant aboveground biomass and soil C accumulation under these meadows.

## Introduction

In grassland ecosystems, soils represent the largest active pool of organic C which may have global implications [1]–[3]. In N-limited grasslands, N deposition through precipitation and/or dust accumulation or fertilization and sometimes P fertilization can increase the productivity of the plant community but also can decrease species richness [2], [4]–[9]. However, N and P fertilization may affect soil C fractions and the ecological function of grassland soils in different ways [2], [10]. Long-term N fertilization can decrease microbial biomass carbon (MBC) of grassland soil [11], [12]. Fertilization effects on C mineralization are variable [13]–[15]. Mack et al. [16] showed that NP fertilization contributed to decreases in soil organic C (SOC) pools in tundra ecosystems. Phosphorus additions can lead to greater C sequestration in soils [17]. In addition, N additions can significantly enhanced soil C stocks, but combined NPKMg additions may not result in soil C sequestration [9]. Thus, it is clear that in contrast to the consistent increases of productivity due to N and P fertilization, the impacts of N and P fertilization on microbial composition, activity and soil C is not consistent and differs markedly between sites.

Alpine grasslands in the Qinghai-Tibetan Plateau are estimated to hold approximately 55% of China's total grassland C, out of which 93% is present in the soil [18]. Little is known about how fertilization types and rates affect soil C fractions in alpine meadows.

Here we specifically examined how 5 years of N and/or P additions had influenced soil organic C fractions. Our objective was to develop a better understanding of the effects of N and P fertilization on changes in soil organic C dynamics. Previous studies from the same experiment have shown that N or N+P fertilization increased plant aboveground productivity and there was a yearly variation in plant biomass [19]–[21]. Forbs and legumes richness decreased after N and P fertilization [21]. Nitrogen concentration and N/P ratios of grasses and forbs increased significantly but were relatively constant for legumes after N addition [22], [23]. In this paper, we divided species into different functional groups to address the changes in soil organic C fractions in relation to the species changes. Specifically, we evaluated the effects of N and P fertilization on the aboveground plant biomass of different functional groups and soil C fractions at 0–20 cm and 20–40 cm.

Fertilization can affect the quantity of C input to soil by alteration of the plant productivity, diversity, and stoichiometry of plant tissues [16], [24], as well as soil organic matter decomposition and respiration by altering soil microbial communities and activity [11], [25]. We hypothesized that a combination of N and P fertilization will increase SOC but decrease MBC, microbial functional diversity and C mineralization rates compared to N and P fertilization alone or no fertilization.

## Methods

### Ethics Statement

Our study area was located at the Alpine Meadow Ecosystem Research Station in Hezuo, Gansu, eastern Qinghai-Tibetan Plateau, China (N34°55', E102°53' 3,000 m above sea level), which is managed by Lanzhou University. No specific permissions were required for conducting experiment in this location, and no endangered, protected species or vertebrate species were involved with this research.

### Study site

This study was conducted from 2009 to 2013 in the Research Station of Alpine Meadow and Wetland Ecosystems of Lanzhou University, located in Hezuo, Gansu, eastern Qinhai-Tibetan Plateau of China (N34°55', E102°53', 3,000 m above sea level). Hezuo has a 30-year mean annual precipitation of 550 mm, with 85% of the precipitation occurring during the growing season from June through September (Institute of Hezuo Meteorology). Mean annual temperature is 2.4°C, ranging from −8.3°C during December-February to 11.9°C during June–August periods. The soils are classified as chestnut soils or Haplic Calcisols according to the FAO classification or sub-alpine meadow soil according to the Chinese soil classification system [26]. The vegetation is a typical alpine meadow, dominated by grasses such as *Festuca ovina* Linn., *Poa poophagorum* Bor and *Elymus nutans* Griseb; sedges such as *Scirpus pumilus* Vahl and *Kobresia capillifolia* (Decne.) C.B. *Kobresia pygmaea* C.B. Clarke in Hook; forbs such as *Anemone rivularis* Buch.-Ham, *Trollius farreri* Stapf and *Anemone obtusiloba* D. Don, *Taraxacum lugubre*, *Geranium pylzowianum*, *Polygonum viviparum*, and legumes such as *Astragalus polycladus* Bur., *Oxytropis ochrocephala* and *Gueldenstaedtia verna* Georgi.

### Experimental design

Soil organic C fractions resulting from the increased plant production by N and P fertilization were evaluated using the following fertilization treatments: (i) N alone; (ii) P alone; and (iii) N+P together. There were three rates (5, 10, 15 g m^−2^ yr^−1^) of N (urea with 46% N), and P (sodium dihydrogen phosphate anhydrous with 44.6% P). A control treatment (CK) without any N or P fertilization was also included. As a result, there were ten treatments (N_5_, N_10_, N_15_, P_5_, P_10_, P_15_, N_5_P_5_, N_10_P_10_, N_15_P_15_, and CK) with five replicates for a total of 50 treatment plots. Only four replicates were used in this study. All treatments were randomly assigned in 50 plots (5×5 m area each). In addition, there were four plots without fertilization but destroyed by zokors (*Myospalax fontanieri*) in 2009, but this didn't affect the neighbor plots. All plots were separated by 1 m unfertilized buffers. Fertilizer was broadcast evenly in the plot once per year on 20^th^ August for 5 years since 2009. Fertilizer was applied in August for two reasons: (i) In our study site, the growing season was from June through September and 85% of the rainfall occurred during the growing season. (ii) Plant biomass peaked at this time, which was reasonable to be contrasted with that before fertilization application. However, applying fertilizers in the middle of the growing season might result in the inefficient use and wastage. Before applying fertilizers in August 2009, average SOC, soil total N and total P was 33.8±1.8 g kg^−1^, 3.7±0.1 g kg^−1^, 0.65±0.01 g kg^−1^ in 0–20 cm and 26.1±1.5 g kg^−1^, 2.8±0.1 g kg^−1^, 0.65±0.01 g kg^−1^ in 20–40 cm, respectively, across all plots. The soil had 200 g kg^−1^ sand, 600 g kg^−1^ silt, and 200 g kg^−1^ clay.

### Plant sampling

In early August 2013 before applying fertilizers, four sampling quadrates of 50 cm×50 cm (selected at random from the central of each 5×5 m plot to avoid edge effects) were used to harvest plant samples at 1 cm above the ground level using scissors. Plant samples were separated into three functional groups: grasses, legumes, and forbs. At the same time, root samples were collected also within the quadrates after clipping the aboveground plant. Four soil cores (5 cm diameter by 20 cm depth) in each quadrate were collected randomly using a hand probe and composited by plot. Soil samples were washed gently with water over a 60 mesh screen until roots were separated from the soil. All above- and belowground plant samples were dried at 65°C to constant mass and ground to 1 mm prior to determination of total C and N. Aboveground plant samples were also collected from 2009 to 2012, oven dried, ground, and C and N concentrations determined as above.

### Soil sampling

In addition to samples collected for root biomass, soil samples were also collected for determining C fractions. Five cores (5 cm in diameter) in 0–20 cm and 20–40 cm depths were taken randomly in each plot, and mixed into one composite sample by depth. After removing gravel, coarse fragments and roots, each soil sample was homogenized and divided into two portions. One portion was air dried and sieved to 2 mm to analyze abiotic parameters. The other portion was kept at 4°C for analysis of microbial parameters (soil MBC, functional diversity of microbial community, and C mineralization).

### Plant and soil analysis

Plant C and SOC concentrations were determined using the dichromate oxidation method [27]. Plant and soil total N was determined following Kjeldehl digestion by a Nitrogen Analyzer System (KJELTEC 2300 AUTO SYSTEM II, Foss Tecator AB, Sweden).

Soil MBC was determined by using the chloroform fumigation extraction method [28]. Field-moist soils were adjusted to 50% water holding capacity, incubated at 25°C for 2 weeks for uniform rewetting and stabilizing the microbial activity after the initial disturbances. Three 25 g sub-samples were fumigated with alcohol-free CHCl_3_ for 24 h at 25°C. Samples were then extracted for MBC by adding 100 ml of 0.5 M K_2_SO_4_, shaking for 60 min and filtered through Whatman No. 2 paper. Three 25 g non-fumigated soil sub-samples were processed in the same manner. Carbon concentration in the extract was determined using the same method as SOC and MBC was calculated as the difference in C concentrations between fumigated and non-fumigated samples divided by the efficiency factor 0.38 [28].

Soil incubation for C mineralization was carried out in the laboratory for 28 days. Dry weight equivalent of 30 g of the sieved field-moist soil samples were weighed into 250 ml Schott jars. Small beakers filled with 15 ml of 1 M NaOH were placed at the soil surface in the jars to trap the evolved CO_2_. The jars were fastened airtight and incubated for 28 days at 25°C. Moisture content of the samples was periodically adjusted to a value of 30% of water holding capacity since this is generally considered to mimic the moisture content of the field conditions [29]. Constant soil water content was maintained by weighing each sample once a week and adding water to the soil. The CO_2_ evolved from the soil was measured at 1, 7, 14, 21, and 28 day after incubation. After each day of incubation, the small beaker with NaOH solution was removed and replaced by a new one with fresh NaOH. The CO_2_ absorbed in NaOH solution was titrated with 0.1 M HCl after the addition of BaCl_2_. A NaOH solution without soil, incubated as above, was also titrated. Basal respiration was calculated as the amount of CO_2_ evolution in the first 24-h incubation divided by the dry mass of soil. The metabolic quotient (*q*CO_2_) was calculated as the ratio of basal respiration to MBC [30]. The cumulative C mineralization was calculated as the sum of carbon dioxide (CO_2_) released during 28 days of incubation.

### Bacterial functional diversity in Biolog EcoPlate

Field-moist soils were used to assess the functional diversity of the microbial community, measured by Biolog EcoPlate system [28], [30]–[32] according to the procedure describes by Li et al [33]. Average well color development (AWCD) in each microplate was determined as described by Garland [34]. AWCD and Shannon-Wiener diversity index in Biolog EcoPlate were calculated using data of Biolog EcoPlate collected during 0–168 h and at 168 h respectively as described by Li et al [33].

AWCD  =  [∑(C-R)/31], C is sum of optical densities of 31 wells in a plate, R is optical density of control well in the same plate.

Shannon-Wiener diversity H  =  -ΣP_i_lnP_i_, of which P_i_ is proportional color development of the i^th^ well over total color development of all wells of a plate.

### Data analysis

Data were analyzed using SPSS 16.0 and graphs were plotted using Sigma Plot 10.0. In this paper, biomass data for only in the fifth year (i.e. 2013) was analyzed because data for the first four years have already been published [20], [21]. A one-way ANOVA was used to analyze data by considering all treatments (three fertilization types × three rates + unfertilized control  = 10 treatments). When the analysis was significant, the control was taken out and a two-way MANOVA was used to test the overall effects of fertilization types and rates on measured parameters. The least significant difference (LSD) test was used to separate means at *P* = 0.05. Principal component analysis (PCA) was performed on AWCD data at 168 h of incubation in the BIOLOG Ecoplate as described by Zhang et al [35] to determine the carbon utilization patterns of soil microbial communities. Regression analysis was used to analyze the relationship between cumulative carbon mineralization rate at the end of incubation (28th day) and soil MBC and SOC in all treatments. Regression analysis was also used to analyze the relationship between SOC and C:N ratios of different plant functional groups, roots or soils.

## Results

### Fertilization effects on plant aboveground biomass

Both fertilization type and rate influenced plant community biomass, grass and legume biomass, with a significant type × rate interaction for grass and community biomass (Table 1).

**Table 1:** Univariate tests: ANOVAs on plant biomass of community and three functional groups: grasses, legumes and forbs, and on soil microbial biomass C (MBC), *q*CO_2_, cumulative C mineralization, soil organic C (SOC) in 0–20 cm and 20–40 cm for fertilization types and rates.

	Plant biomass				MBC		*q*CO2		Cumulative C mineralization		SOC	
	Community	Grasses	Legumes	Forbs	0–20	20–40	0–20	20–40	0–20	20–40	0–20	20–40
Type	0.000	0.000	0.717	0.468	0.003	0.004	<0.001	0.073	<0.001	<0.001	0.049	0.016
Rate	0.018	0.001	0.663	0.009	<0.001	<0.001	0.016	0.003	0.573	0.693	0.807	0.009
Type* Rate	0.018	0.002	0.762	0.357	<0.001	<0.001	<0.001	0.118	0.031	0.503	0.095	0.052

**Figure 1:**
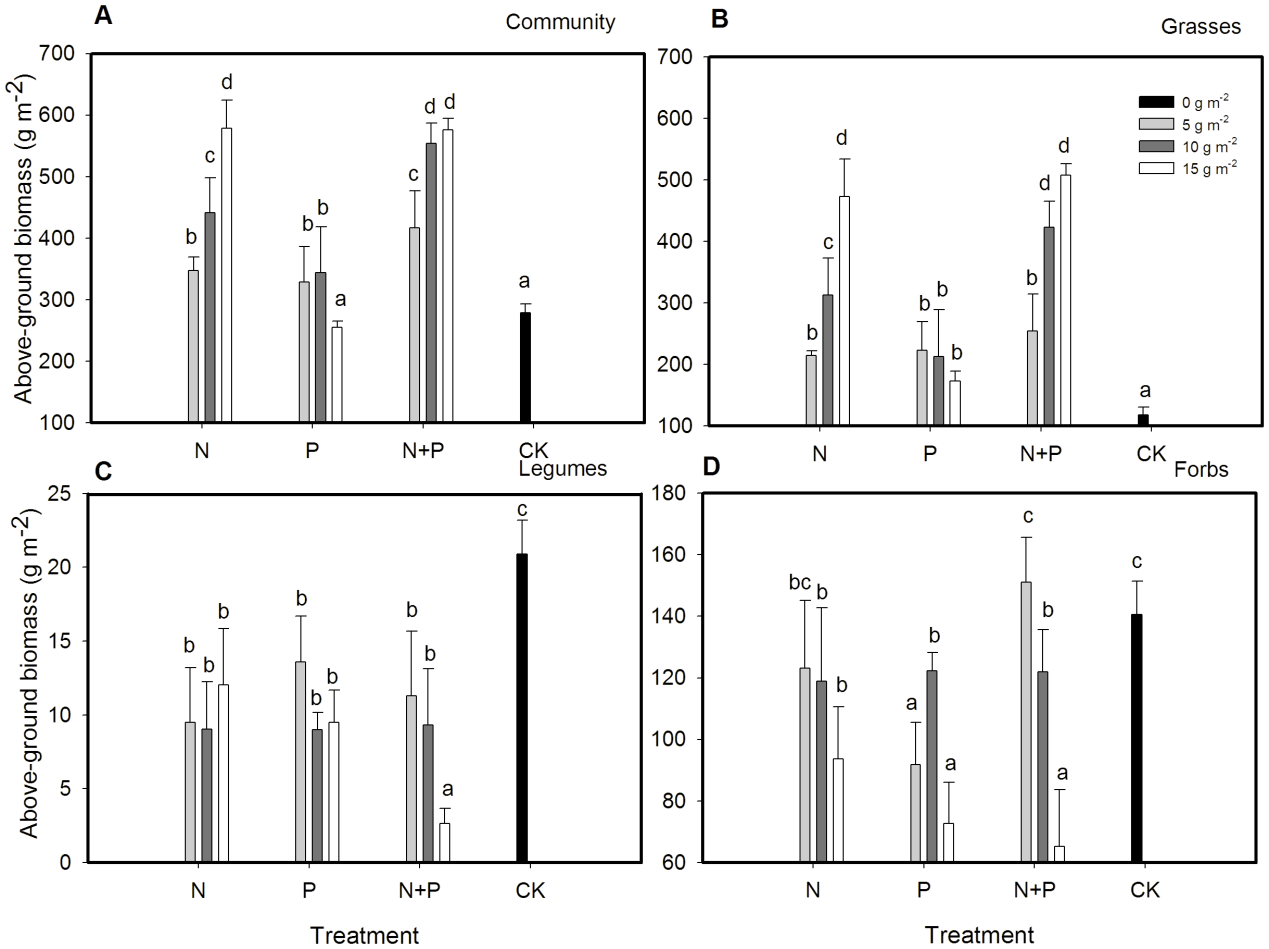
Mean ± SE of total aboveground plant biomass of (A) community, (B) grasses, (C) legumes, and (D) forbs as affected by N and P fertilization treatments. Different letters above bars indicate significantly different at *P* = 0.05.

Eight of nine fertilization treatments (except the 15 g m^−2^ yr^−1^ P fertilization) had higher community biomass than the control (Figure 1A). Combination of N+P at 5 g m^−2^ yr^−1^ and 10 g m^−2^ yr^−1^ had much higher community biomass than corresponding rates of N fertilization or any rates of P fertilization. Nitrogen or N+P at 15 g m^−2^ yr^−1^ had two times community biomass and four times grass biomass compared to the control (Figure 1A, B). Grasses comprised 70% of the total biomass, and its biomass trend with fertilization was similar to that of total biomass. All N+P rates had higher grasses biomass compared to corresponding N fertilization rates although no difference occurred between 15 g m^−2^ yr^−1^ N+P and 15 g m^−2^ yr^−1^ N. The N+P treatments at 10 and 15 g m^−2^ yr^−1^ had much higher grass biomass as compared to the corresponding P fertilization rates (Figure 1B). However, the biomass of legumes and forbs in all fertilization treatments (except forbs in 5 g m^−2^ yr^−1^ of N+P fertilization) were lower than the control (Figure 1C, D). The N+P fertilization at 15 g m^−2^ yr^−1^ had significantly lower legume and forb biomass than other treatments, except the forb biomass at 15 g m^−2^ yr^−1^ of N and P.

### Fertilization effects on carbon/nitrogen ratio of plant and soil

**Figure 2:**
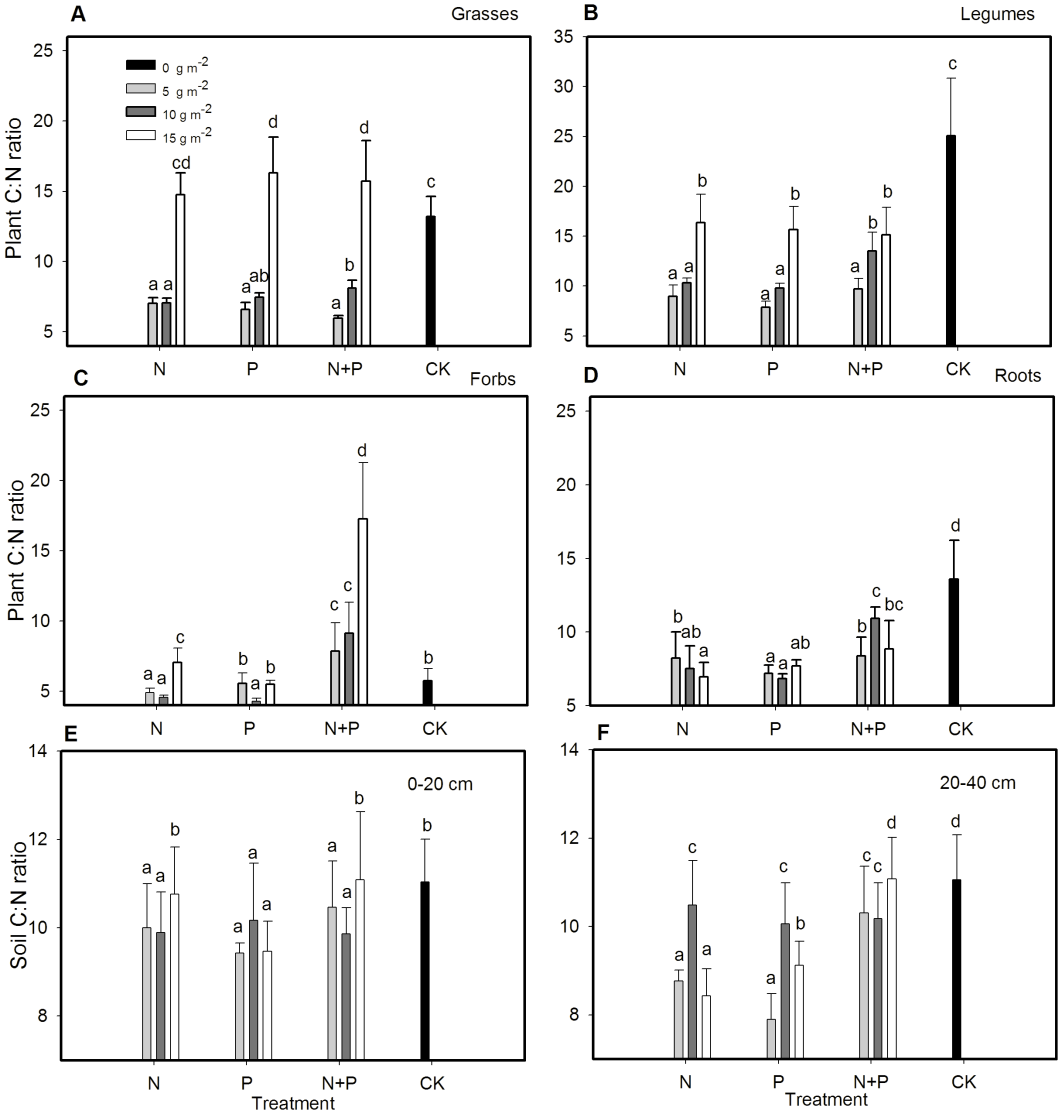
Mean ± SE of C:N ratios of (A) grasses, (B) legumes, (C) forbs, and (D) roots, (E) 0–20 cm soil, (F) 20–40 cm soil as affected by N and P fertilization treatments. Different letters above bars indicate significantly different at *P* = 0.05.

Fertilization type influenced C:N ratios of the different plant functional groups. Fertilization rate and the fertilization type × rate interaction were significant for forb C:N ratio (Table 2). All fertilization at 5 and 10 g m^−2^ yr^−1^decreased whereas fertilization at 15 g m^−2^ yr^−1^ increased grasses' C:N ratios compared to the control (Figure 2A). The N, P, and N+P rates decreased C: N ratios of legume and root (Figure 2B, 2D). The 5 and 10 g m^−2^ yr^−1^ N fertilization and 10 g m^−2^ yr^−1^ P decreased while N+P at all rates increased forbs' C:N ratios (Figure 2C). Fertilization type had significant effect on soil C concentrations in both layers and fertilization rate had significant effect on soil C concentrations in 20–40 cm. However, neither fertilization type nor fertilization rate had significant effects on soil N concentrations and C:N ratios in both layers (Table 2). Nevertheless, soil C:N was lower in fertilization treatments (except in 15 g m^−2^ yr^−1^ of N or N+P in 0–20 cm and 15 g m^−2^ yr^−1^ of N+P in 20–40 cm) than the control (Figure 2E, 2F).

**Table 2:** Univariate tests: ANOVAs on C:N ratios of plant (different functional groups and roots) and soil in 0–20 cm and 20–40 cm for fertilization types and rates

	Plant C:N ratio				Soil C:N ratio	
	Grasses	Legumes	Forbs	Roots	0–20 cm	20–40 cm
Type	<0.001	<0.001	<0.001	0.381	0.626	0.058
Rate	0.152	0.474	<0.001	0.695	0.807	0.169
Type* Rate	0.095	0.676	<0.001	0.390	0.890	0.346

### Effect of fertilization on soil carbon fractions

Fertilization type and rate had significant effects on soil MBC, *q*CO_2_, cumulative C mineralization, and SOC at 0–20 cm and 20–40 cm layers, except for the effect of rate on C mineralization at both layers and SOC at 0–20 cm (Table 1). The fertilization type × rate interaction was significant for all parameters in all layers, except for *q*CO_2_ and C mineralization at 20–40 cm.

**Figure 3:**
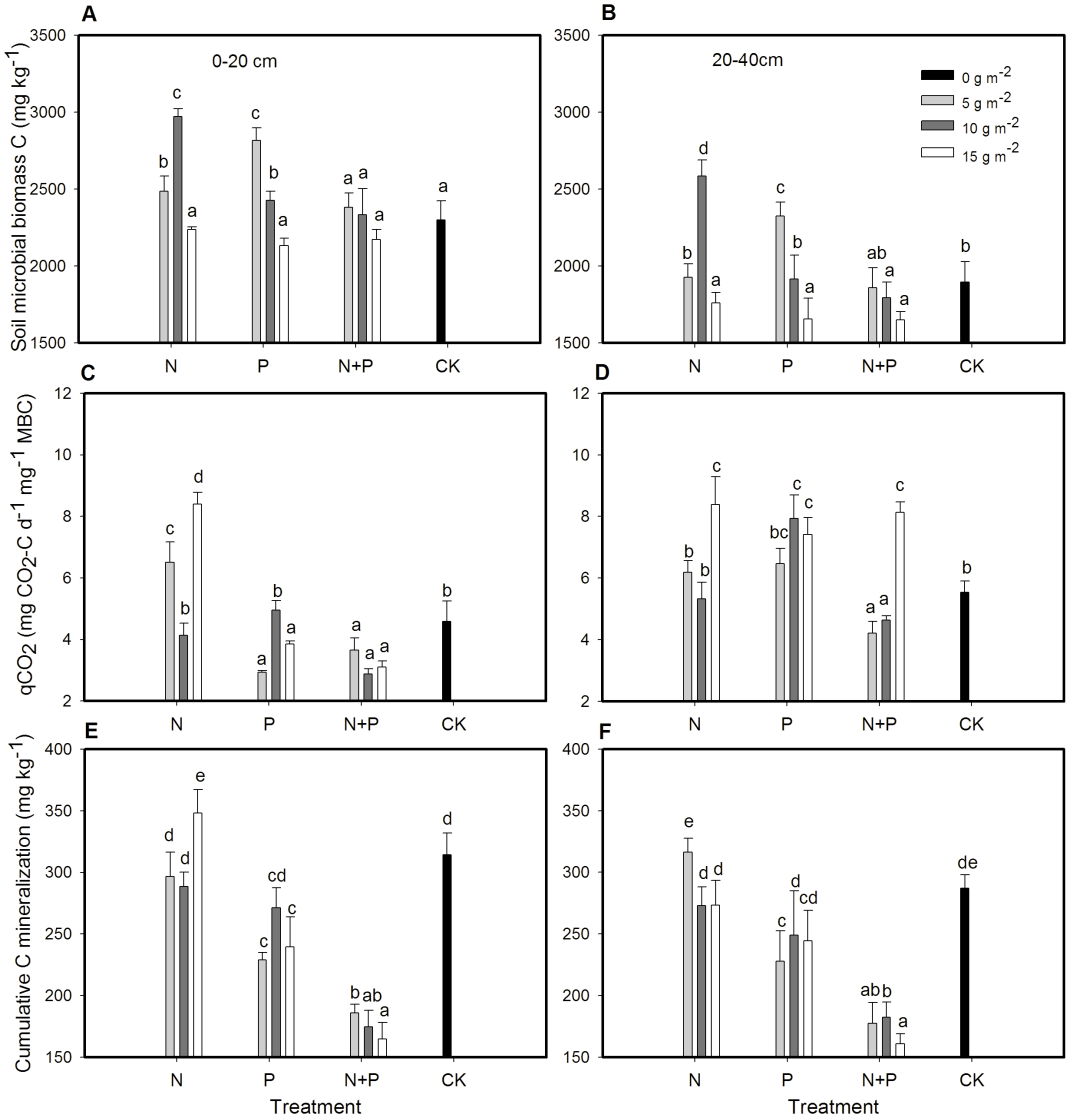
Mean ± SE of (A) and (B) soil microbial biomass C, (C) and (D) metabolic quotient (*q*CO_2_), (E) and (F) cumulative C mineralization in 0–20 cm and 20–40 cm soil layers as affected by N and P fertilization treatments. Different letters above bars indicate significantly different at *P* = 0.05.

Nitrogen and P at rates of 5 and 10 g m^−2^ yr^−1^ increased MBC compared to other treatments in 0–20 cm (Figure 3A). In 20–40 cm, 10 g m^−2^ yr^−1^ of N fertilization and 5 g m^−2^ yr^−1^ of P fertilization significantly increased MBC compared to other treatments (Figure 3B). Soil MBC was significantly lower in 5 and 10 g m^−2^ yr^−1^ of N+P than those in corresponding rates of N or P fertilization in both layers (Figure 3A, B).

Nitrogen fertilization at 5 and 15 g m^−2^ yr^−1^ significantly increased *q*CO_2_ while P fertilization at 5 and 15 g m^−2^ yr^−1^ and N+P at all rates significantly decreased *q*CO_2_ in 0–20 cm (Figure 3C). The N+P at 5 and 10 g m^−2^ yr^−1^ decreased *q*CO_2_ compared to other treatments in 20–40 cm (Figure 3D).

Most of the fertilization treatments (except the 15 g m^−2^ yr^−1^ N in 0–20 cm soil and 5 g m^−2^ yr^−1^ N in 20–40 cm soil) had lower cumulative C mineralization rates than the control. All N+P fertilization rates had lower cumulative C mineralization than other treatments (Figure 3E, F).

**Figure 4:**
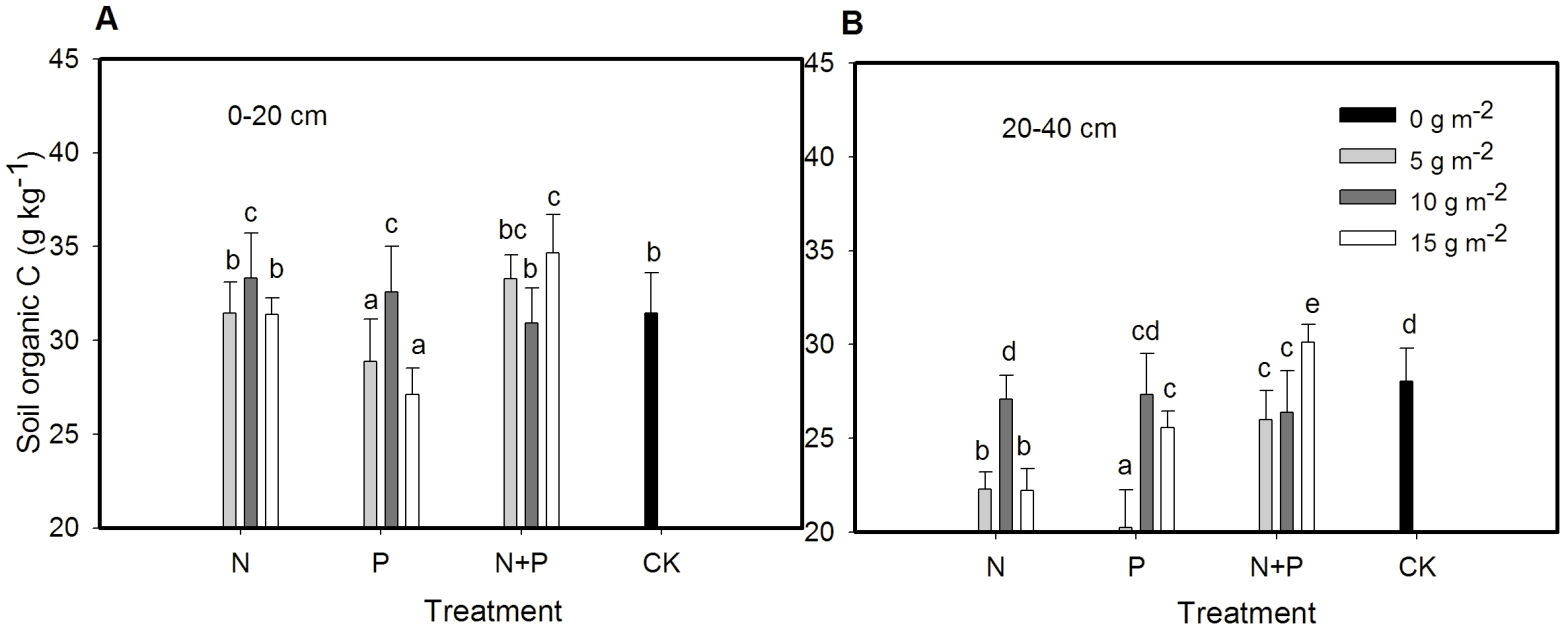
Mean ± SE of soil organic C in 0–20 cm (A) and 20–40 cm (B) as affected by N and P fertilization treatments. Different letters above bars indicate significantly different at *P* = 0.05.

Soil organic C averaged across treatments was higher in 0–20 cm than 20–40 cm (Figure 4A, B). Compared to the control, 10 g m^−2^ yr^−1^ of N or P fertilization, and 15 g m^−2^ yr^−1^ of N+P fertilization significantly increased SOC in 0–20 cm (Figure 4A). The SOC at 20–40 cm was greater in 15 g m^−2^ yr^−1^ of N+P than other treatments (Figure 4B).

### Effect of fertilization on functional diversity of soil microbial community

**Figure 5:**
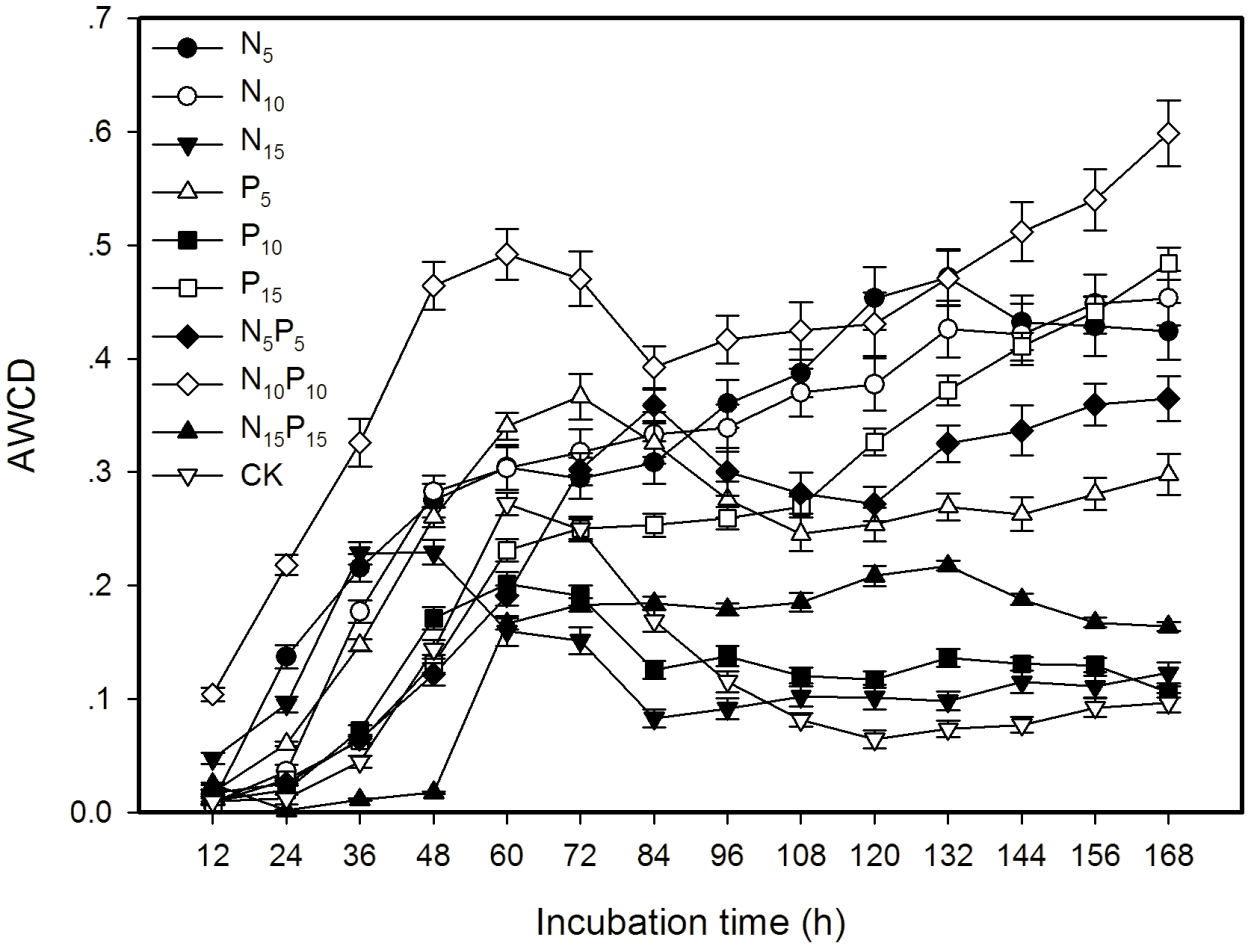
Average well color development (AWCD) (means ± SE) of soil microbial community in BIOLOG Ecoplate as affected by N and P fertilization treatments.

**Figure 6:**
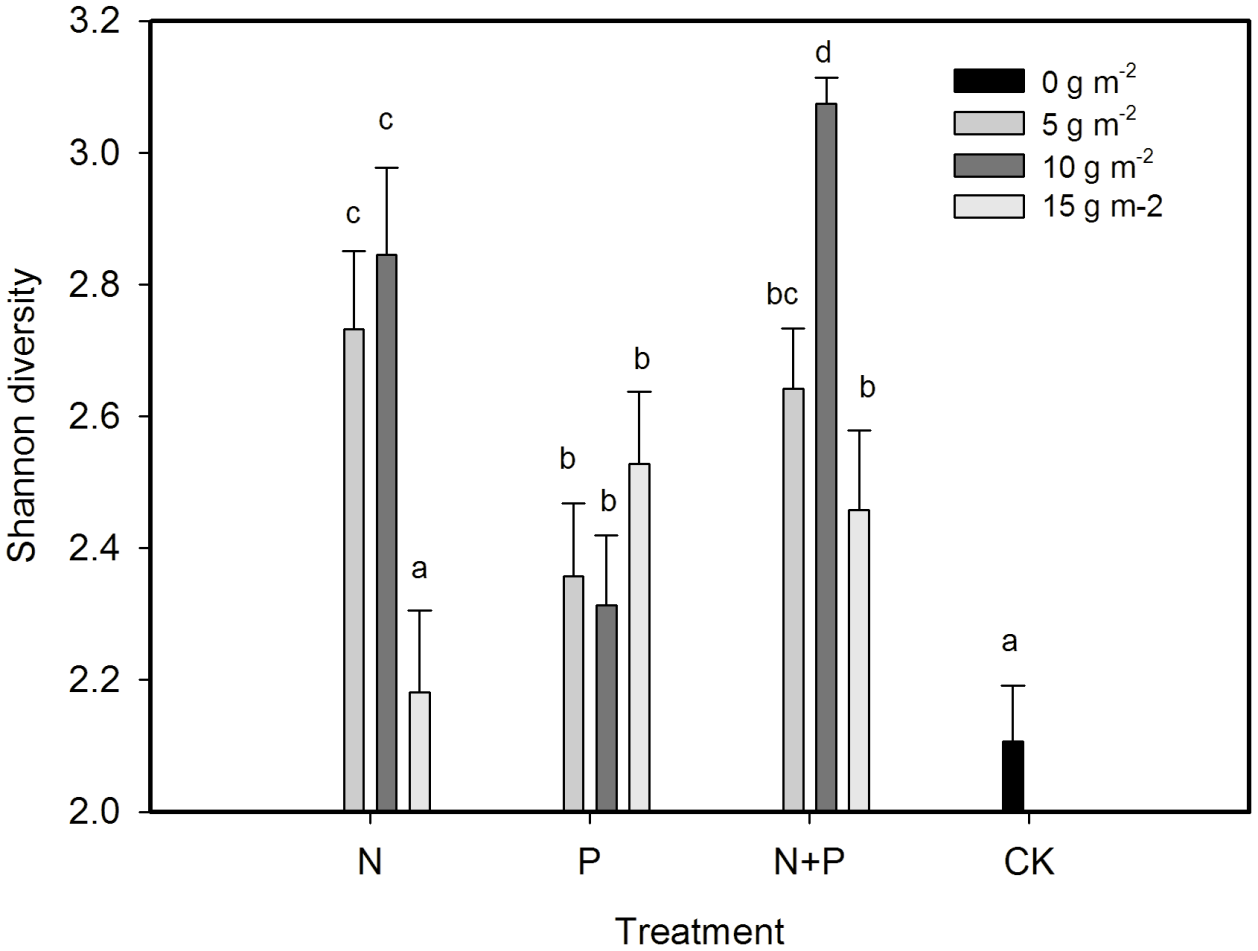
Functional diversity of soil microbial community in 0–20 cm as affected by N and P fertilization treatments Different letters above bars indicate significantly different at *P* = 0.05.

The AWCD was usually higher in 10 g m^−2^ yr^−1^ of N+P application throughout the incubation than other treatments (Figure 5). The AWCD was lower in N+P than N fertilization at 5 g m^−2^ yr^−1^, but it was higher in N+P than N fertilization at 10 and 15 g m^−2^. Eight of nine fertilization treatments (except 15 g m^−2^ yr^−1^ N) significantly increased the Shannon functional diversity of soil bacterial community compared to the control (Figure 6). The N+P fertilization at 10 g m^−2^ yr^−1^ had the highest diversity, followed by 10 and 5 g m^−2^ yr^−1^ N fertilization

**Figure 7:**
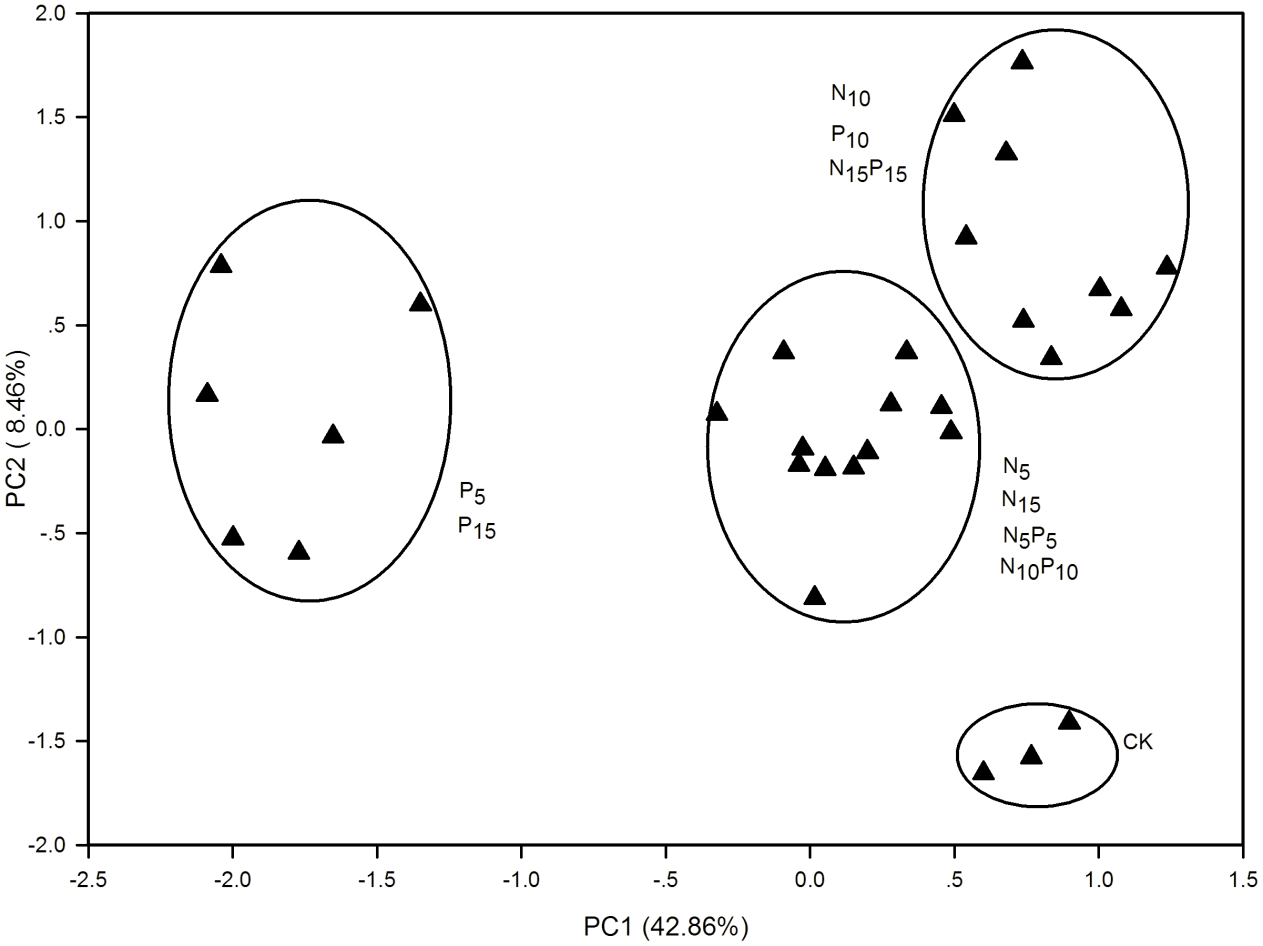
Principal component analysis results according to average well color development data of carbon substrate groups at 168 h in BIOLOG Ecoplates. N, nitrogen fertilization; P, phosphorus fertilization; CK, unfertilized control; subscript numbers 5, 10, 15 indicate corresponding fertilization rates (g m^−2^ yr^−1^).

In the PCA ordination diagram, samples with similar AWCD datasets were located close to one another, and those dissimilar were located far apart (Figure 7). The PCA results showed that the C substrate utilizing profiles were separated into four distinct groups: 1) 5 and 15 g m^−2^ yr^−1^ of P fertilization; 2) 10 g m^−2^ yr^−1^ of N or P fertilization, 15 g m^−2^ yr^−1^ of N+P fertilization; 3) 5 and 15 g m^−2^ yr^−1^ of N fertilization, 5 and 10 g m^−2^ yr^−1^ of N+P fertilization; and 4) unfertilized control. Metabolic profiles from the treatments within each group were similar to each other, but significantly different from other groups. The first two PCs (PC1 and PC2) explained 42.86% and 8.46% of the variance in AWCD data. The main loadings on the PC1 axis were carboxylic acids (0.87), polymers (0.83), amino acids (0.79), amine/amides (0.78), and carbohydrates (0.75), and on the PC2 axis were amino acids (0.77), and carboxylic acids (0.59).

## Discussion

### Response of plant biomass, functional group composition, plant and root C:N ratio to fertilization

Fertilization probably increased the growth and biomass of plants, especially grasses, by supplying essential nutrients, such as N and P [21]. The N and P-induced increase in plant biomass was consistent with previous study [21] and N addition experiments in other grasslands [4]–[9]. Previous study has shown that N and N+P fertilization significantly decreased species richness but N+P addition significantly increased species number of grasses, and species loss was mainly due to the loss of forbs and legumes after fertilization [21]. Studies from other grasslands also have shown that N addition significantly decreased species diversity [5]–[9]. Fertilization-induced increase in aboveground plant productivity and community coverage resulted in a decrease of light penetration in community canopy. This resulted in reduced growth of legumes and forbs under intense light competition, leading to local extinction of these species [21], [36], [37].

Fertilization-induced decrease in C:N ratios of all plant functional groups and roots was consistent with previous studies in alpine meadows that N concentrations of grass and forbs increased significantly thereby decreasing C/N ratios while these of legumes were relatively constant after N addition [22], [23]. This might be due to the species specific ecological stoichiometry [38]. Grasses had higher N and P use efficiency than other functional groups, which resulted in increase in N concentration after fertilization [38]. The height and biomass of forbs didn't change or even decreased. Legume species were not sensitive to N additions for they could get additional N by N-fixing [22], [38]. Studies from other grasslands also showed that N inputs consistently increased soil N availability, leading to tissue and litter N-enrichment in semiarid shrublands [15], [39].

### Response of soil microbial community to fertilization

Increase soil MBC in N or P fertilization treatments was contrary to our hypothesis and inconsistent with some studies that showed long-term N addition decreased MBC by 20–35% [25], [40]. However, in our study site, N fertilization rate was much lower than those in other studies conducted in Europe and North America [25], [40]. Increase in aboveground plant biomass and in turn C input to soil following N and P fertilization might be responsible for the observed increase in soil MBC. The increase in carbon substrate to microbes and alleviation of microbial N or P-limitation could increase the microbial growth [41], which was responsible for the increase in AWCD and functional diversity after fertilization in our study. Reduction in microbial biomass and activity with P and N+P fertilization might be due to the toxicity of nutrients that affected MBC and favored one species of microbe over another [42]. However, increased N availability could stimulate carbon-degrading enzyme activities [43], [44] and microbial enzyme shifts could result in increased C mineralization and *q*CO_2_ [46].

Our study showed similar patterns of *q*CO_2_ and C mineralization with fertilization treatments, but inverse to the pattern of MBC. The decrease in C mineralization might be due to a decline in lignin degradation induced by reduction enzyme activity and/or fungal population densities, and modification of the soil microbe composition following fertilization [45]–[47]. Ramirez et al. [40] reported that there were consistent phylum-level changes in the bacterial communities across soils with the N-amended soil. The N-induced shifts in microbial community structure should yield corresponding shifts in the functional and metabolic potentials of the communities, resulting in a change in decomposition rates [40]. In our study, we did not measure soil microbial composition or enzyme activities; however the results of C substrate utilization data based on Biolog Ecoplate indicated that N, P and N+P fertilization could increase microbial functional diversity and affect microbial carbon utilization and metabolism. Increased rates of N or P fertilization alone increased microbial biomass but reduced their activity, probably due to favorable growth of certain microbes over another due to fertilization. This was likely the negative impact on microbes by decreasing soil pH [42] and increasing toxicity [43] following N fertilization.

The combination of N+P fertilization however increased AWCD and Shannon diversity. This might be due to the following reasons: (i) Alleviation of microbial N and P-limitation and the alteration of community composition, resulting in increased growth in certain microbial population [48]. (ii) Changes in plant community structure and composition which affected soil microbial community. (iii) Grass roots provided unique attachment sites for certain microbial populations, e.g. mycorrhizal fungi, or grass growth stimulated the formation of certain microbial communities after fertilization [49]. This was supported by principal component analysis of carbon utilization data which revealed that microbial functional diversity depended on the plant community components. These findings suggested that fertilization may alter the structure and composition of soil microbes and modify substrate utilization patterns by soil microbes in the alpine meadow, resulting in a change in C mineralization rates.

### Soil organic C

Our data showed that plant community biomass was positively correlated with SOC in 0–20 cm (R^2^ = 0.115, P = 0.032). The increased soil organic C only appeared in 10 g m^−2^ yr^−1^ of N, P and 15 g m^−2^ yr^−1^ of N+P compared to control. The increase in soil organic C agreed with previous studies, which showed that N and P additions significantly enhanced C stocks and C sequestration in grassland soils [34], [49]–[51]. In our study, plant community biomass increased and grasses accounted for 70% of the total biomass in eight of nine fertilization treatments. So, it was the amount of C input returned mostly from grasses that increased SOC. Also C:N ratio was higher in grasses than forbs in two of the three fertilization treatments. Since 15 g m^−2^ yr^−1^ of N+P fertilization increased grass biomass, the increase in SOC with this treatment was most likely to be due to increased C input and slower decomposition of grasses due to higher C/N ratio. These findings suggested that 15 g m^−2^ yr^−1^ of N+P fertilization can be used to sequester C in such alpine meadow soils. The decrease in soil organic C with 5 and 15 g m^−2^ yr^−1^ of P in 0–20 cm and most of the fertilization treatments (except 15 g m^−2^ yr^−1^ of N+P) in 20–40 cm agreed with several studies which have found that N and P fertilization contributed to decreases in soil organic C pools in tundra ecosystems [16], [45]. In our study, SOC concentration was positively correlated with C:N ratios of grasses (R^2^ = 0.112, P = 0.035 in 0–20 cm soil and R^2^ = 0.261, P = 0.001 in 20–40 cm soil respectively) and forbs (R^2^ = 0.124, P = 0.026 in 0–20 cm soil and R^2^ = 0.185, P = 0.006 in 20–40 cm soil respectively), suggesting that the decrease in soil organic C concentration was partially due to changes in plant diversity and productivity of functional groups and decrease in plant C:N ratios, in particular the dominant functional groups—grasses and forbs. Given that soil represented the largest active C pool in terrestrial ecosystems, and, in particular, alpine meadows with higher soil organic C [52], a decrease in SOC and C accumulation might lead to an increase in CO_2_ emission to atmosphere.

After 5 years of fertilization, decreased SOC in 5 and 15 g m^−2^ yr^−1^ of P in the upper soil layer and most of the fertilization treatments (except 15 g m^−2^ yr^−1^ of N+P) in the deeper soil layer indicated that soils with these fertilization treatments in these meadows will not be a strong C sink, and that N or P fertilization may even lower the soil organic C pool. Combined with the increase in plant community productivity and changes in the C:N ratios of plant tissues, our results also suggested that a new balance between C sequestration by vegetation and soil organic C mineralization will determine whether alpine meadow ecosystems become a C sink, source or vary across space and time. A study from European grassland showed that multi-nutrient (N, P, K, Mg) additions increased aboveground plant productivity (APP), and N+P had higher APP than N alone, but lower APP than NPKMg fertilization [9]. However, soil C sequestration was increased by N-only additions, not multi-nutrient fertilization [9]. An experiment from our study site showed that APP increased with the increasing number of added limiting resources (N, P, K, and water) and APP was higher in N+P treatment than in N+P+K treatment or N, P alone treatment [19]. Therefore, in alpine meadows, both N and P fertilization need to be applied at adequate rates (15 g m^−2^ yr^−1^) to increase C sequestration, not N or P alone. Potassium combination with N and P might not further increase plant biomass according to study of Ren et al. [21], but further studies were needed to examine their effects on SOC in alpine meadows.

### Conclusions

Nitrogen alone and N+P fertilization at rates of 15 g m^−2^ yr^−1^ doubled aboveground biomass of plant communities, driven by a fourfold increase in grass biomass. The combination of N and P fertilizers increased SOC but reduced microbial biomass and activity and C mineralization compared to N or P alone or no fertilization. The N+P at rate of 15 g m^−2^ yr^−1^ can be used in increasing aboveground biomass of plant community and especially grass biomass and dominance, resulting in increased soil C accumulation at the surface layer in these alpine meadows.
